# The Plethora of Microbes with Anti-Inflammatory Activities

**DOI:** 10.3390/ijms25052980

**Published:** 2024-03-04

**Authors:** Flora Tsvetanova

**Affiliations:** Institute of Chemical Engineering, Bulgarian Academy of Sciences, 1113 Sofia, Bulgaria; florablue@abv.bg

**Keywords:** anti-inflammatory activity, microorganisms, lactic acid bacteria, fungi, marine bacteria

## Abstract

Inflammation, which has important functions in human defense systems and in maintaining the dynamic homeostasis of the body, has become a major risk factor for the progression of many chronic diseases. Although the applied medical products alleviate the general status, they still exert adverse effects in the long term. For this reason, the solution should be sought in more harmless and affordable agents. Microorganisms offer a wide range of active substances with anti-inflammatory properties. They confer important advantages such as their renewable and inexhaustible nature. This review aims to provide the most recent updates on microorganisms of different types and genera, being carriers of anti-inflammatory activity.

## 1. Introduction

In the last decades, inflammation has turned into the main reason causing various diseases in human organisms. Inflammation is an immunological defensive response to external stimuli such as tissue injury, microbial pathogen infection, or chemical irritation and is the basis of a variety of physiological and pathological conditions [1]. This biological process includes the innate and adaptive immune system. Inflammation is initiated by the migration of immune cells from blood vessels and the release of mediators, followed by the accumulation of inflammatory cells and the secretion of reactive oxygen species (ROS), reactive nitrogen species (RNS), and pro-inflammatory cytokines for the inactivation of the pathogens and the repair of the injured tissue [2]. Various ROS and RNS, like O_2_^−^, OH, H_2_O_2_, NO, and O_2_, produced by the cellular biomolecules that arise from inflammatory cell damage (nucleic acids, proteins, lipids) are reflected in the augmentation of the state of inflammation [2]. The complex body response includes leukocyte cells, macrophages, neutrophils, and lymphocytes [3]. As a result of the inflammation, these cells release special substances, including amines and vasoactive peptides, eicosanoids, pro-inflammatory cytokines, and acute phase proteins, which mediate the inflammatory process and prevent additional tissue damage [4]. The activation of macrophages plays an important role in the initiation and distribution of the inflammatory response through the production of cytokines such as interleukin 1β (IL 1β), NO, cyclooxygenase-2 (COX-2), tumor necrosis factor-alpha (TNF-α), and other inflammatory mediators [5]. Nuclear factor-kappa B (NF-kB), a nuclear transcription factor, takes part in the regulation of the expression of these cytokines [6].

In grave cases of inflammation, the excessive production of cytokines results in a condition called a “cytokine storm”, which leads to damaging consequences, organ failure, and mortality [7]. During the cytokine storm, the level of IL-6 is three times higher than in normal conditions [8]. The cytokine storm is reported to be the major reason for patients’ death from COVID-19 [9].

Acute inflammation is a short-term self-limiting condition and the host’s defenses can easily return the body to homeostasis. However, chronic inflammation provokes chronic disease development characterized by infiltration of inflammatory cells, excessive production of cytokines, dysregulation of cellular signaling, and loss of barrier function [10]. Chronic inflammation is a crucial component in multiple progressive diseases and conditions such as neurological disorders (Alzheimer’s and Parkinson’s diseases), chronic inflammatory diseases (chronic obstructive pulmonary disease, psoriasis, pancreatitis, inflammatory bowel disease), hypertension, cancer, cardiovascular diseases (heart failure, atherosclerosis, cardiomyopathy, coronary diseases), type 2 diabetes, metabolic disorders (fatty liver disease, chronic kidney disease), rheumatoid arthritis, and osteoporosis [2]. Diverse medicinal products are being developed worldwide as a counteraction to inflammation in the human body. These drugs can be divided into three groups—corticosteroids, non-steroidal anti-inflammatory medicines, and disease-modifying anti-rheumatoid drugs. Although these chemicals contribute to the reduction in inflammatory symptoms, they can exhibit side effects concerning the gastrointestinal tract, including bleeding, platelet dysfunction, etc. [11]. Therefore, it is very important to employ natural products that on the one hand are not toxic to the human organism, and on the other hand, are available, renewable, and inexhaustible. Such opportunities are given to us by the plants and microorganisms that live in or around us.

**Figure 1:**
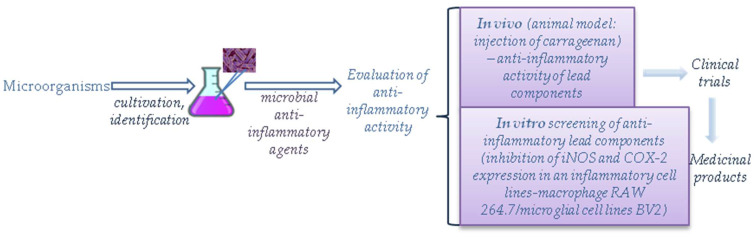
Common steps in the evaluation of the anti-inflammatory activity of selected microorganisms.

Drug research and development is a long-lasting, costly, and rigorous process that requires the screening of an enormous number of substances and serious investment. This pathway includes target selection, validation, and lead discovery [12]. During validation, animal or human primary cells, cell lines, patient explants, or humanized animal models of a relevant disease are employed. The final major step of early drug discovery is the lead discovery process where the lead compound can come from various sources including microorganisms [13]. Usually, the structural modification of the lead is necessary to improve its selectivity, potency, and pharmacokinetic parameters [14]. The lead compound should then undergo detailed in vivo assay for activity, toxicity, and efficacy establishment, the so-called preclinical studies, followed by the clinical studies [13]. Figure 1 demonstrates the steps from the isolation of microbes with anti-inflammatory activity to the drug development.

This review targets the microbes that are carriers of anti-inflammatory activity. It summarizes species of different types and different habitats—from those living inside and around us to those living in the deep sea.

## 2. Human Microbiota and Human-Friendly Microbes with Anti-Inflammatory Properties

Human microbiota refers to the trillions of commensal microorganisms that are colonizing our body. Microbes living in immediate relationships with humans (on different surfaces of the body) are very important for protection against infections and diseases [15,16]. Normally, skin flora consists of representatives of bacteria (*Propionibacterium*, *Staphylococcus*, and *Corynebacterium* species), fungi (*Malassezia* spp., *Aspergillus* spp., *Cryptococcus* spp., *Rhodotorula* spp., *Epicoccum* spp., etc.), and viruses [17]. The gut microbiome is an ecological entity characterized by thousands of microbes including bacteria, viruses, and some eukaryotes. The most dominant phyla (comprising up to 90% of gut microbiota) are Bacteroidetes and Firmicutes (including *Lactobacillus* sp.) followed by Proteobacteria, Fusobacteria, Tenericutes, Actinobacteria, and Verrucomicrobia [18].

Human skin microbiota play an important role in skin health maintenance and the potential prevention of premature skin aging. Utilization of probiotics in skin therapeutic products is an attractive idea as it can provide interesting alternative possibilities for some skin disorders. In their study, the team of Khmaladze compared the employment of a live culture and lysate of *Lactobacillus reuteri* DSM 17938, aiming to investigate the anti-inflammatory and barrier functions of skin in ex vivo models and in vitro assays for antimicrobial activity. They induced inflammation through the application of ultraviolet B radiation in the human epidermis and native skin models. The levels of IL-6 and IL-8 decreased in both assays—with live culture and lysate. It was established that live *Lactobacillus reuteri* increased the expression of the aquaporin 3 gene while the lysate enhanced laminin A/B levels in a healthy reconstructed human epidermis which suggests a positive impact on the skin barrier. It can be concluded that *Lactobacillus reuteri* DSM 17938 could be used in skin products to avoid the UVB-R-mediated inflammatory cascade and/or to prevent photoaging, to enhance barrier function, or for unhealthy skin prone to inflammatory outbreaks [19].

In a recent study, Brandy et al. demonstrated that lactic acid bacteria (LAB) lysates stimulated the proliferation of keratinocytes and that *L. plantarum* SGL 07 and *L. salivarius* SGL 19 hastened the skin’s re-epithelialization through the induction of keratinocyte migration. The secretion of specific pro-inflammatory mediators from keratinocytes was also reduced. In addition, it was shown that a specific proteome modulation of the exposed keratinocytes was induced involving protein dysregulation (interleukin enhancer-binding factor 2 and an ATP-dependent RNA helicase) and pathways (such as cytokine, NF-kB, Hedgehog, and RUNX signaling) associated with the anti-inflammatory activity. These results indicate that these strains together could be used in therapies for skin relief [20].

*Streptococcus salivarius* inhabits the oral cavity and the digestive tract. This strain becomes a normal inhabitant of the human body just a few hours after birth and remains predominant. In experiments, it exerted anti-inflammatory activity in vitro as it inhibited the activation of the NF-kB pathway on intestinal epithelial cells. The performed mouse model experiments resulted in significantly reduced inflammation in severe and moderate colitis [21]. According to Kaci and collaborators, the in vitro anti-inflammatory effects could be a result of the intracellular signaling pathway and the innate immune responses of epithelial cells rather than of the immunocompetent cell-based host immunity [21]. *Streptococcus salivarius* has been used in New Zealand as a probiotic for prevention of streptococcal pharyngitis and halitosis [22].

*Rothia mucilaginosa* is one more normal human commensal of the oral cavity that has exhibited anti-inflammatory activity. It is also found in the lower respiratory tract during chronic diseases. Two types of assays with this bacteria were performed—in vitro (three-dimensional cell culture model) and in vivo (mouse model)—resulting in the conclusion that *R. mucilaginosa* expresses an inhibitory effect on the pathogen- or lipopolysaccharide-induced pro-inflammatory responses. It was illuminated that *R. mucilaginosa* inhibited the production of pro-inflammatory cytokines by lung epithelial cells in vitro and induced high IL-8 production caused by the pathogen *P. aeruginosa*, and moderate or low IL-8 production in the case of exposure to *S. aureus* SP123, *S. anginosus* LMG14696, *A. xylosoxidans* LMG 26680, and *G. haemolysans* LMG 18984. The anti-inflammatory effect of *Rothia* was confirmed in a 3D model of cystic fibrosis epithelial cells. The LPS-induced pro-inflammatory response in an in vivo model was lowered. The supernatant of *Rothia* inhibited IL-8 production and NF-kB pathway activation in response to a pro-inflammatory stimuli [23]. The invention of anti-inflammatory activity in *Rothia* in the lung microbiota leads to the hypothesis that other nonpathogenic inhabitants in the respiratory tract may possess immunomodulatory properties.

Inflammatory bowel diseases (IBDs) are conditions associated with the chronic abnormal inflammation of the gastrointestinal tract during which levels of IL-8 cytokines increase [24] and ROS are overproduced [25]. The two basic types of IBDs are Crohn’s disease (CD) and ulcerative colitis (UC) with different physical symptoms displayed [26]. The core of these conditions is the disturbed balance between microorganisms in the gut [27]. In general, the studies with CD and UC patients reveal the reduced presence of Firmicutes and Bacteroidetes phyla thus enhancing levels of Gammaproteobacteria [28,29]. The dysbiosis results in changes in the production of short-chain fatty acids, which in turn can affect the inflammatory pathways and immune system modulation [30]. *Christensenella minuta* DSM 22607 is a commensal bacteria usually found in the human gut and is believed to be maintaining the microbial balance there. The absence of *C. minuta* is related to IBDs. Its anti-inflammatory effect was tested on human intestinal lines where reduced levels of pro-inflammatory IL-8 cytokines via the inhibition of the NF-kB signaling pathway were achieved. This strain was also shown to protect intestinal epithelial integrity in vitro. More in-depth, the strain was proven to prevent intestinal damage, reduce colonic inflammation, and promote mucosal healing in two distinct animal models of acute colitis. Both the supernatant and the bacterial culture were confirmed to possess anti-inflammatory properties. The supernatant decreased NF-kB pathway activation by 40%; however, when the bacteria alone was used, no anti-inflammatory effects were monitored. Therefore, *C. minuta* probably secretes a potent anti-inflammatory effector into the supernatant [31]. Its anti-inflammatory capacity was also reported in the study of Relizani and co-workers, where colonic inflammation was reduced through the inhibition of the NF-κB signaling pathway and the secretion of proinflammatory cytokines IL-8 and IL-1β [32]. Moreover, a decrease in the concentration of LCN-2 (a non-invasive biological marker of intestinal inflammation) was registered in animal models where treatment with *C. minuta* was applied [33].

The deficiency of *Faecalibacterium prausnitzii* is also related to CD. *Faecalibacterium* can account for up to 6.5% of the human microbiota [34]. The supernatant of this bacteria is known to exert anti-inflammatory effects both in vivo and in vitro. Quévrain et al. identified the nature of the peptide with anti-inflammatory properties produced by *F. prausnitzii*. This 15 kDa protein—MAM (microbial anti-inflammatory molecule)—managed to inhibit the NF-kB pathway in intestinal epithelial cells and to prevent colitis in an animal model. This work opens new lines for the prevention and treatment of IBD as MAM could represent a targeted biomarker for CD in terms of *F. prausnitzii* for predicting CD relapse [35]. Another representative, *Faecalibacterium duncaniae*, was observed to enhance the levels of a specific subset of IL-10-secreting Treg cells located in the lamina propria of the colon. These cells are missing in patients with IBDs [36]. Lactobacilli have been demonstrated to impede inflammatory processes via different mechanisms, one of them, for example, is the maintenance of the balance of intestinal microbiota. Experiments with animal models of the IBD pathologies demonstrated the relationship between LAB administration with beneficial modulations of the intestinal microbiota, and more specifically, with the normalization of lactobacilli levels, and the reduction in *Clostridium perfringens*, enterococci, and coliforms [37]. This beneficial effect was also confirmed in human trials [38]. LAB can act by reducing the oxidative stress related to IBDs, as well [39].

*Bacteroides thetaiotaomicron* is a gut commensal bacteria that produces extracellular vesicles responsible for its anti-inflammatory activity. These vesicles were administered to mice treated with colitis-inducing dextran sodium sulfate. Ameliorated symptoms of intestinal inflammation and improved survival rate were achieved. In addition, a high ratio of IL-10/TNF-α production was observed [40].

## 3. Bacteria and Yeasts with Anti-Inflammatory Properties in Food

Microbes are on one side responsible for food contamination and spoilage, and on the other—they are at the core of obtaining beneficial products that make a major contribution to the food industry. Yeast and bacteria are the most widely used microorganisms in the food industry. They produce different foods through fermentation, the main contributor among bacteria being lactic acid bacteria (LAB) [41].

**Figure 2:**
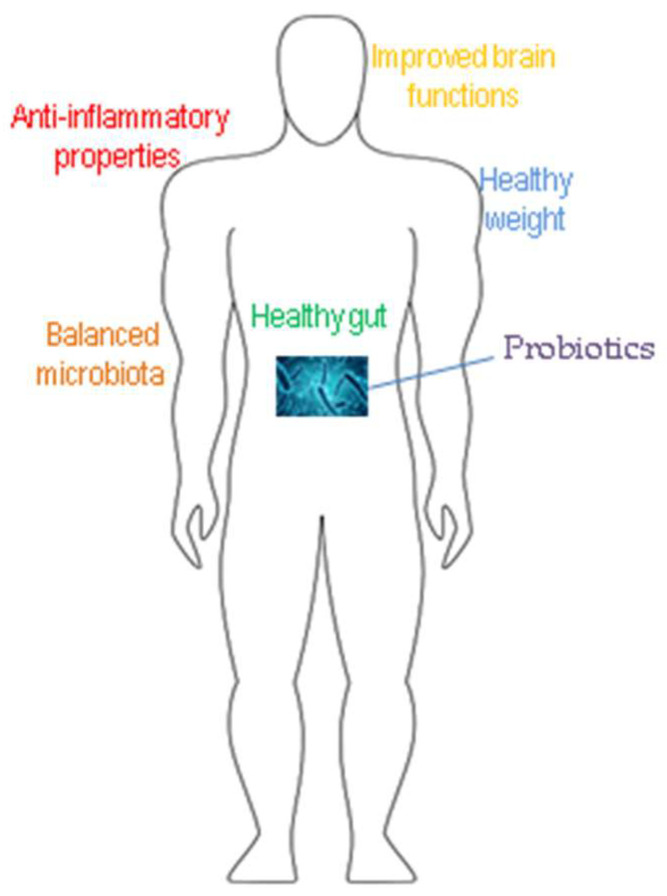
Main beneficial effects of probiotics.

LAB represent a heterogeneous group of microorganisms from different genera—*Lactobacillus*, *Enterococcus*, *Carnobacterium*, *Lactococcus*, *Leuconostoc*, *Pediococcus*, *Oenococcus*, *Streptococcus*, *Vagococcus*, *Tetragenococcus*, and *Weissella*. Some strains of LAB together with several strains from *Bifidobacterium* create probiotics. Probiotics are defined as “live microorganisms that when administered in adequate amounts confer a health benefit to the host” [42]. They have been traditionally used as starter cultures for the preparation of fermented foods where lactic acid is the main product of carbohydrate metabolism. Lactic acid is known to inhibit the growth of many pathogens. LAB are included in functional foods as they provide multiple benefits for the host organism and possess GRAS status. LAB exert immunomodulating effects through the stimulation of IL-10 production and/or decreasing pro-inflammatory cytokines [43]. There is plenty of evidence demonstrating the beneficial role of specific lactobacilli strains in inflammatory conditions including wound healing, skin protection, defense against pathogenic agents, etc. [44,45,46,47,48]. Figure 2 illustrates the beneficial effects of LAB on the human organism. However, to manifest their beneficial qualities, they should be able to survive in the gastrointestinal tract and be present in adequate quantities [49]. The probiotic bacteria possess the ability to adhere to the host‘s intestinal mucosa, which reveals its relationship with gut mucosa cells and the generated immunomodulatory effect on the host, and also the pathogen competitive exclusion [50]. Moreover, a study from 2021 demonstrated that the probiotic lactobacilli ameliorate the symptoms of inflammation that occur as a result of anti-inflammatory drugs. The administration of probiotics to mice led to an increase in the population of anaerobes and lactobacilli along with a serious decrease in the total enterobacteria. Therefore, probiotic supplementation in non-steroidal anti-inflammatory drug-induced inflammation increased the intestinal antimicrobial activity, thus maintaining intestinal homeostasis [51]. In another work, the team of Santiago-Lόpez highlighted the potential of *Lactobacillus fermentum* to improve indomethacin (a non-steroidal anti-inflammatory drug)-induced inflammation [52]. These examples indicate that the cited lactobacilli possess even stronger anti-inflammatory effects than the non-steroidal anti-inflammatory drugs.

Besides the production of lactic acid, LAB produce various beneficial substances including B-group vitamins (riboflavin, folates, cobalamin, thiamine), bacteriocins, bioactive peptides, biosurfactants, flavoring compounds, etc. Different strains of lactobacilli with anti-inflammatory activity are given in Table 1.

### 3.1. Vitamins-Producing LAB

Riboflavin (vitamin B12) plays a major role in cellular metabolism as it is a precursor of flavin mononucleotide and adenine flavin dinucleotide, coenzymes that protect cells from ROS. Unbalanced ROS secretion is usually associated with chronic intestinal inflammation in the initial stages of IBDs. Several studies have demonstrated the anti-inflammatory potential of riboflavin which plays the role of an antioxidant enzyme cofactor. For example, in the study of Al-Harbi and co-workers, vitamin B12 intake inhibited the production of TNF-α [72]. On the other hand, its short-term deficiency was found to affect the ability of macrophages to induce adequate immune response while its supplementation reduced the macrophage pro-inflammatory activation [73]. This vitamin was also demonstrated to decrease the inflammation caused by oxidative stress in diabetes [74]. Riboflavin-producing *Lactobacillus plantarum* CRL2130 was reported to reduce intestinal inflammation in a model of colitis in mice through the control of pro-inflammatory cytokines. For the aims of the experiment, the strain was wrapped in a food matrix (fermented soymilk), or administered as a bacterial suspension [56,75]. All these studies show that riboflavin could be an adequate source of prevention of inflammation.

Except for riboflavin, folates and folic acid (vitamin B9) also take part in the reduction and prevention of inflammation. A study from 2017 explored the relationship between arterial hypertension and the decrease in serum homocysteine concentrations, and indirectly with the decrease in other marks of inflammation [76]. Pan and collaborators reported that the reason for IBDs may be associated with folate deficiency [77]. Moreover, folic acid supplementation could decrease the risk of colorectal cancer development in IBD-suffering individuals [78]. *Lactobacillus reuteri* ATCC PTA6475 indicated the ability to suppress the production of TNF—α in human monocytes—and exerted anti-inflammatory activity in a murine model of acute colitis. This quality is determined by the folC2 gene involved in folate biosynthesis. [53].

A probiotic blend of LAB-producing vitamins was described by Levit et al. The strains *Lactobacillus plantarum* CRL2130, *Streptococcus thermophilus* CRL807, and *Streptococcus thermophilus* CRL808 with immunomodulatory properties were studied for anti-inflammatory activities. The results displayed even more emphasized anti-inflammatory effects than in the case of individual strains administered to rodents with induced intestinal mucositis [63]. The successful application of microbial mixtures regarding anti-inflammatory effects was reported for *Lactobacillus casei*, *Lactobacillus plantarum*, *Lactobacillus acidophilus*, *Lactobacillus delbrueckii* subsp. *bulgaricus*, *Bifidobacterium longum*, *Bifidobacterium breve*, *Bifidobacterium infantis*, and *Streptococcus salivarius* where the tumor development associated with intestinal inflammation in mice was prevented [64]. These conclusions indicate that the employment of blends of microbes could enhance their individual anti-inflammatory properties.

In general, vitamin-producing LAB, especially those that secrete riboflavin and folate, could find application in the treatment of various diseases. Additionally, the blends of vitamin-producing LAB would not only enhance the efficiency of the primary treatments but would also fill the shortages of essential vitamins provoked by the disease or the treatment [79]. According to Albuquerque et al., vitamin production can be initiated by prebiotic supplementation [80]. A very important invention accessed in murine models, is that the vitamins produced by LAB are biologically active [81,82].

### 3.2. Anti-Inflammatory Exopolysaccharides (EPSs) Producing Bacteria

Another substance with proven anti-inflammatory properties secreted by LAB, especially by *Lactobacillus kefiranofaciens*, is the exopolysaccharide kefiran, which is a branched gluconolactone. Kefiran is a constituent of the fermented beverage kefir. The consortium of microbes in kefir include the bacterial strains *Lactobacillus*, *Lactococcus, Leuconostoc, Streptococcus*, *Acetobacter*, and the yeasts *Kluyveromyces, Torula*, *Candida*, and *Saccharomyces*. According to Jenab et al., kefiran induced both CD^4+^ and CD^8+^ T-lymphocyte populations [65]. In another study, IL-4 and IL-5 levels were decreased to normal amounts after its administration in the ovalbumin-induced BALB/c mice asthma model. Therefore, kefiran has the potential to be used for pulmonary inflammatory treatment [66]. It is believed to be a promising candidate for the treatment of bronchial asthma and lung inflammation [83].

Besides the kefir consortium, symbiotic microorganisms of the Tibetan mushroom were also revealed to possess anti-inflammatory properties. This symbiotic population consists of bacteria and yeast living in polysaccharide grains secreted by them. Among the organisms of the consortia are *Lactobacillus casei*, *Lactobacillus acidophilus*, *Lactococcus lactis*, *Leuconostoc citrovorum*, *Leuconostoc mesenteroides*, *Acetobacter aceti*, *Acetobacter rasens*, *Streptococcus thermophilus*, *Streptococcus lactis*, *Kluyveromuces* sp., and *Sacharomyces* sp. [84]. The Tibetan mushroom suspension was tested on a cotton-induced granuloma and paw edema in rats resulting in a significant inhibition of 43% in the formation of granuloma tissue [67].

Exopolysaccharides isolated from the cell surface of the putative probiotic organism *Lactobacillus paraplantarum* BGCG11 were tested in a rat model induced by carrageenan injection in the hind paw. Reduced expression levels of pro-inflammatory mediators IL-1β, TNF-α, and iNOS, and enhanced levels of pro-inflammatory cytokines IL-10 and IL-6 were reached. This suggests that the antihyperalgesic and antiedematous effects of EPSs were associated with inflammatory response suppression [59]. EPSs from *Lactobacillus rhamnosus* RW-9595M were detected to induce higher levels of TNF-α, IL-6, and IL-12 though inhibited IL-10 production [60].

*Bacillus licheniformis* BioE-BL11 and *Leuconostoc mesenteroides* BioE-LMD 18 isolated from the Korean fermented kimchi were found to inhibit the secretion of pro-inflammatory cytokine IL-6 in the LPS-stimulated RAW 264.7 mouse macrophage. In addition, the secretion of the anti-inflammatory cytokine IL-10 was increased and it was established that the extent of the inhibition was dose-dependent. These results suggest that EPSs produced by *B. licheniformis* and *L. mesenteroides* could be proper ingredients for application in pharmaceutical products, cosmetics, and in the food industry [69]. In another study, *L. plantarum* LM17, *L. plantarum* LM19, and *L. rhamnosus* LM07 with probiotic characteristics were isolated from the agave fermentation stage in mezcal production [70]. The strains were shown to exert anti-inflammatory properties in TNF-α-stimulated HT-29 cells. These species were tested in vivo in a mouse model with dinitrobenzene sulfonic acid (DNBS)-induced chronic colitis. The inflammatory indicators were weight loss, intestinal permeability, and cytokine profiles. *L. plantarum* improved mice health as observed by reduced weight loss and significantly decreased intestinal permeability. These results confirmed the capability of LAB isolated from the agave fermentation to be used as probiotic supplements for IBD treatment [70]. The study of Hsieh and Allen manifested that the exopolysaccharides from the probiotic strain *Bacillus subtilis* protected mice from acute colitis caused by the pathogen *Citrobacter rodentium*. The polysaccharides inhibited the activation of T-cells and therefore controlled T-cell-mediated immune responses in multiple inflammatory diseases [85].

### 3.3. Biosurfactants with Anti-Inflammatory Properties-Producing Bacteria

Biosurfactants are natural products derived from bacteria, yeasts, or fungi, and represent amphiphilic compounds that contain polar-(water soluble) and non-polar-(insoluble in water) sections. The amphiphilic structure of biosurfactants is important for surface tension reduction. This property is applied in various industries such as pharmaceutical, cosmetics, food, and petroleum [86]. Biosurfactants are produced when the carbon source is an insoluble substrate; thus, microorganisms facilitate their diffusion into the cell by the production of different substances. They reduce surface tension in bacteria. Glycolipids, a polysaccharide–lipid complex, phospholipid, mycolic acid, lipoprotein, or lipopeptide are the possible structures of biosurfactants. Besides the availability of insoluble carbon sources, biosurfactant production may be induced by the culture conditions, such as temperature, pH, agitation, and concentration of P, Fe, N, Mg, and Mn ions in the media [87]. Among the biosurfactant-producing bacteria are *Bacillus*, *Rhodococcus*, *Mycobacterium*, *Pseudomonas*, *Arthrobacter*, etc. [88]. *Bacillus subtilis* produces the cyclic lipopeptide surfactin. Surfactin possesses several biological activities among which is its anti-inflammatory action. Its mechanism of action in the lipopolysaccharide (LPS)-stimulated macrophages displayed prevention of the secretion of anti-inflammatory agents (such as IL-1β and iNOs), and reduction in TNF-α and NO levels in response to septic shock [89]. The central LPS receptor is TLR4. It is a signal transduction pathway mediating LPS-induced inflammation. The studies have shown that surfactin downregulated the LPS-induced TLR4 protein expression of macrophages. Surfactin was also demonstrated to express anti-inflammatory activity by reducing the activation of nuclear factor-kB, which plays a role in the NF-κB cell signaling pathways [89,90]. Zhao et al. reported a *Bacillus subtilis* with anti-inflammatory properties owing to the possession of the cyclic lipopeptides, fengycin and iturin [90].

In conclusion, microbial biosurfactants possess various advantages over chemical ones; for example, they are less toxic, have higher foaming capability, have higher biodegradability, and exhibit specific activity at extreme pH and temperature [88].

### 3.4. Bacteriocins with Anti-Inflammatory Properties—Producing Lactobacilli

Lactobacilli produce bacteriocins, extracellular compounds with peptide structures synthesized by ribosomes. Their function is the defense against other microorganisms [91]. One example of a bacteriocin produced by lactobacilli is nisin which enhances the bacterial activity of mononuclear phagocytes. This action occurs through the increase in autophagy-inducing cytokine-like IFN-γ levels and decreasing IL-4 and IL-13 which is followed to downregulate the lung Th2 response, which is known to restrict autophagy. Therefore, therapy with probiotics could modulate the immune responses in the lung which augment the regulatory T-cell response in the therapy of peripheral blood mononuclear cells (PBMCs) and macrophages with combined *M. tuberculosis* and LAB [92]. Bacteriocins produced by *Lactobacillus rhamnosus* successfully reduced IL-6 and C-reactive protein levels in the serum accumulated after infectious diseases. Therefore, these biochemicals could be used in the prevention of postoperative infections [61].

### 3.5. Other Substances with Anti-Inflammatory Effects Produced by Lactobacilli

LAB are known to produce γ-aminobutyric acid (GABA). GABA is a non-protein amino acid that regulates neuronal activity by nerve transmission inhibition. Its deficiency is associated with various neurological diseases, such as epilepsy, anxiety, depression, Alzheimer’s and Parkinson’s diseases, Huntington’s chorea, and schizophrenia [93]. According to several studies, GABA enhances plasma growth, the brain’s protein synthesis, hormone level, cognitive ability and memory, relaxes nerves, and lowers blood pressure [94,95]. The advantage of microbially produced GABA over a chemically synthesized one is that it is economically safe and friendly [55]. Soltani et al. reported that GABA increased the production of the anti-inflammatory mediator TGF-β1 and decreased the production of inflammatory mediators such as IL-β1, TNF-α, interferon-γ, and IL-12 in streptozotocin-treated mice [96]. Kim et al. isolated GABA-producing LAB from several fermented Korean foods and evaluated the anti-inflammatory effects of these strains on macrophages to determine their potential utilization as probiotics. The strains with pronounced GABA-producing ability were from the species *L. brevis*. The analysis revealed that almost all of the strains inhibited NO and iNOs production, and NF-kB activity, thus exhibiting anti-inflammatory activity [55]. Therefore, these GABA-producing lactobacilli can act as probiotics that control nerve excitement.

Kombucha, a fermentative drink, and the consortium involved in its production were the subject of an anti-inflammatory assay [68]. Kombucha, which is a beverage of Manchurian origin, is the fermentation product of various bacteria (*Lactobacillus* sp., *Acetobacter xylinoides*, *Gluconobacter oxydans*, *Komagataeibacter xylinum*, *Gluconacetobacter hansenii*, *Oenococcus oeni*, *Komagataeibacter europaeus*) and yeast strains (*Schizosaccharomyces pombe*, *Zygosaccharomyces kombuchaensis*, *Torulaspora delbrueckii*, *Saccharomyces* sp., *Brettanomyces* sp., etc.). In kombucha fermentation, the black tea leaves turn into valuable metabolites such as vitamins (C, B1, B2, B12), glucuronic and acetic acids, and ethanol [97]. The fermented drink was analyzed for different biological activities. The anti-inflammatory capacity against the enzyme lipoxygenase (LOX) was measured and it was defined as the percentage of inhibition of the 5-LOX enzyme. The control was nordihydroguaiaretic acid. The authors reported an improvement in the anti-inflammatory activity after the fermentation with the kombucha consortium (87–91%), while the non-fermented tea obtained 66% or even 0% of inhibition. An IC_50_ value of 9.0 ± 0.0 µg/mL was also registered, which is close to the maximal inhibitory concentration of 7.0 ± 0.2 µg/mL for the natural LOX inhibitor nordihydroguaiaretic acid. An optimization of the operational parameters was conducted, as well. According to the obtained results, the higher surface/height ratio of the fermentation vessel accelerated the fermentation kinetics and the anti-inflammatory properties. The observation reached in the aforementioned research indicates that kombucha extracts could be effective candidates for the development of non-steroidal drugs for the treatment of inflammation [68]. The relationship between the anti-inflammatory activity and the culturing conditions was also demonstrated by another group of scientists [57]. Culturing conditions were found to affect the anti-inflammatory effect of *Lactobacillus plantarum* OLL 2712 (with confirmed anti-inflammatory properties) in murine model cells where the levels of IL-10 and IL-12 were measured with OLL 2712 cells prepared under various culture conditions. The IL-10-inducing activities of OLL 2712 cells differed significantly between the groups at different culture phases, different mediums, temperatures, and pH. The cells in their exponential phase of growth exhibited higher IL-10-inducing activity than those in their stationary phase [57].

Metabolites of LAB isolated from equid milk were reported to exhibit anti-inflammatory activity. Kostelac and co-workers isolated two strains of *Lactobacillus* species, namely *L. plantarum* M2 and *L. plantarum* K09, from donkey and mare milk, respectively. They determined the TNF-α suppression associated to their extracellular metabolites in lipopolysaccharide (LPS)-stimulated human peripheral blood mononuclear cells (PBMCs). The extracellular metabolites with molecular mass under 2000 Da were found to suppress TNF-α production up to 67% in LPS-stimulated PBMCs. The latter finding confirmed the anti-inflammatory properties of the two lactobacilli strains. Moreover, no cyto/genotoxic effects toward PBMCs were ascertained as a result of the extracellular metabolite activity. Hence, the production of TNF-α in immune cells can be initialized in the presence of lipopolysaccharides, which are a building block of the outer membrane of G-bacteria [58].

In 2021, a team of scientists reported that the membrane vesicles (MVs) from lactobacilli exert anti-inflammatory therapeutic effects [98]. MVs are extracellular spherical particles shed from the cell surface. They are released in the extracellular space and carry parental cell cargo such as enzymes or nuclear acids responsible for the cell’s communication [99]. Additionally, the authors optimized the culture conditions with *L. casei* DSMZ 20011 and *L. plantarum* NCIMB 8826 to obtain an even more emphasized anti-inflammatory effect. MVs from *L. casei* cultured at a pH of 6.5 with agitation resulted in the strongest interleukin-10 release and tumor necrosis factor-α reduction. Concerning the MV from *L. plantarum*, pH = 5 had the most visible effect on the anti-inflammatory activity [98].

Macrophage migration inhibitory factor (MIF) is a homotrimeric protein, a key cytokine responsible for inflammatory progression. This cytokine binds to the CD74 receptor promoting the immune response cascade through the activation of macrophages and T-cells. In this way, it triggers the production of multiple cytokines like TNF, IFN-γ, IL-1β, IL-2, IL-6, IL-8, NO, COX-2, and PGE2 [100,101]. Nyotohadi and Kok aimed to evaluate the anti-inflammatory capacity of a multi-strain extract of LAB, specifically, MIF. The multi-strain extract consisted of *Lactobacillus casei* EMRO 002, *L. casei* EMRO 213, *L. plantarum* EMRO 009, *L. fermentum* EMRO 211, *L. rhamnosus* EMRO 014, *L. bulgaricus* EMRO 212, and *Rhodopseudomonas palustris* EMRO 201. The team intended to study the potential inhibitory effect of the bacterial consortium on the MIF tautomerase activity, the reversibility, and the mechanism of inhibition. MIF tautomerase activity was inhibited with an IC_50_ value of 7.80 ± 1.96 mg/L. It was confirmed that the reaction is reversible [71]. MIF was also reported to inhibit glucocorticoid production which is a basic factor for the anti-inflammatory effects [102]. Therefore, MIF activity inhibition may relieve the inflammation.

Ayyanna et al. tested two probiotic strains (*Lactobacillus mucosae* AN1 and *Lactobacillus fermentum* SNR1) for anti-inflammatory capacity. Two types of experiments were conducted—with encapsulated and unencapsulated strains in rat paw tissues. The strains were in small capsules which released the contents after a prolonged period. They were administered orally. The first group revealed 85 ± 13% of inhibition while the latter revealed 77 ± 25%. The strains exhibited anti-inflammatory cytokine upregulation and pro-inflammatory cytokine downregulation [49].

Five strains of animal origin, including *Lactobacillus reuteri* MG9012(YH9012), *Lactobacillus fermentum* MG9014(YH9014), *Pediococcus pentosaceus* MG9015(YH9015), *Enterococcus faecium* MG9003(YH9003), and *Enterococcus faecium* MG9007(YH9007), were evaluated for potential utilization as probiotics and for their anti-inflammatory effects. The investigation demonstrated that the strains exerted inhibition of NO and inhibited the expression of inducible nitric oxide synthase and cyclooxygenase. These results indicated that the selected strains are appropriate probiotic candidates for animal hosts. However, their further utilization must be confirmed for safety and effectiveness in in vivo investigations [54].

In a study with milk for infant formula, it was confirmed that the anti-inflammatory properties are not related to the inactivated bacteria but to their fermentation products. In vitro and ex vivo experiments with *L. paracasei* CBA L74 were carried out and it was determined that the fermentation products inhibited the pro-inflammatory cytokine secretion leaving anti-inflammatory cytokines either unaffected or even enhanced in response to *Salmonella typhimurium* [103].

The anti-inflammatory properties of whey fermented by *Enterococcus faecalis* M157 against oral cavity pathogens were estimated [104]. Bacterial pathogens cause chronic inflammation leading to periodontitis in the oral cavity, *Porphyromonas gingivalis* being the main causative. Lipopolysaccharides from its cell wall represent the basic virulence factor for chronic periodontitis as they were demonstrated as inducing the production of the inflammatory mediators IL-1β, IL-6, and NO [105]. The M157 strain successfully inhibited IL-1β, IL-6, and induced NO of *Porphyromonas gingivalis* in RAW 264.7 cells. IL-6 and IL-8 in human periodontal ligament cells were also inhibited [104].

## 4. Fungi with Anti-Inflammatory Potential

As an origin of valuable substances and biological activities, fungi are a promising bioresource employed both in the production of functional foods as well as in the pharmaceutical industry [106]. *Talaromyces wortmanii* is an endophytic fungi that was tested for anti-inflammatory activity against the causative of acne vulgaris—*Propionibacterium acnes*. Acne vulgaris is the most common skin complaint leading to serious psychosocial problems such as anxiety and depression. The anaerobic microbe *P. acnes* plays a role in the inflammatory phase of this condition through the activation of the pro-inflammatory mediators IL-8 via the NF-kB and mitogen-activated phosphokinase (MAPK) pathways. As is known, *T. wortmanii* is a producer of bioactive compounds; thus, its crude extract was studied to have the capacity to inhibit the TNF-α-induced ICAM-1 expression and *P. acnes-*induced IL-8 release. Compound C from *T. wortmanii* inhibited the *P. acnes*-mediated activation of NF-kB and AP-1 by means of inhibition of 1 kB degradation and the phosphorylation of ERK and JNK MAP kinases and IL-secretion in a dose-dependent manner. Two compounds were found to be anti-inflammatory substances—BB and C—with an inhibitory activity of 46 and 55%, respectively. However, the mechanism of inhibition remains unclear [107].

A compound named Physcion (C1) was recently isolated from another endophytic fungus, *Aspergillus versicolor* SB5 [108]. This substance exhibited inhibitory activity against COX-2 and LOX-1 with IC_50_ values of 43.10 and 17.54 µg/mL, respectively, making it a potential anti-inflammatory agent [108]. New polyketides from *Aspergillus rugulosa* demonstrated anti-inflammatory activity. Asperulosin A and aspertetronin A significantly inhibited NO production with IC_50_ values of 1.49 ± 0.31 and 3.41 ± 0.85 μM, respectively. In addition, the secretion of anti-inflammatory cytokine IL-10 was visibly enhanced, while the secretion of the pro-inflammatory cytokines IL6, TNF-α, IFN-γ, MCP-1, and IL12 was suppressed. The positive control in the assays was dexamethasone, a glucocorticoid drug used for inflammatory relief. Asperulosin A and aspertetronin A were found to possess better or comparable anti-inflammatory effects in comparison to dexamethasone [109].

Several substances with anti-inflammatory properties were isolated from the endophytic fungus *Colletotrichum gloeosprioides* JS0419. Colletogloeopyrone A, monocillin II, and monocillin II glycoside effectively reduced NO production without cytotoxicity and inhibited the secretion of IL-6 and TNF-α. Monocillin II, and monocillin II glycoside inhibited the protein expression of the NF-κB pathway, inducible NO synthase, and COX-2 while colletogloeopyrone A only inhibited COX-2 expression [110].

Phellinbaumins A and D from the fungus *Phellinus baumii* were found to display moderate inhibitory activity on NO production in a murine model, with IC_50_ values of 31.7 and 24.3 μM, respectively [111]. Two new ergosterol derivatives (chlamydosterols A and B) and three known to have anti-inflammatory activity were isolated from another endophytic fungus—*Fusarium chlamydosporum*. Chlamydosterol A showed a moderate 5-LOX inhibitory capacity with IC_50_ values of 3.06 and 3.57 µM, respectively (compared to indomethacin with an IC_50_ 1.13 µM) [112].

β-glucans in fungi are believed to be one of the sources of its anti-inflammatory properties. β-glucans are polysaccharides, glucose polymers differing based on their length and branching structure. Several studies have demonstrated their beneficial effects on IBDs [113].

## 5. Marine Bacteria and Fungi with Anti-Inflammatory Properties

The ocean represents a rich source of still not fully investigated biological and chemical diversities with potential health benefits. Due to their ocean origin, these microorganisms are an important part of marine ecosystems. They are capable of surviving and reproducing constantly in extreme conditions of low/high pressure, low temperatures, high salinity, oxygen deficiency, and darkness. Besides their ability to survive harsh conditions, marine microorganisms can form symbiotic relationships with other marine organisms, and adapt and evolve at the genetic level. They possess unique metabolic pathways for coping with extreme environmental conditions. Therefore, compared to terrestrial microorganisms, marine ones are more likely to produce secondary metabolites with novel structures and high levels of activity. They are the frontier of new drug discoveries and a large number of bioactives have been derived from them [1,114]. Marine bacteria and fungi are known to produce anti-inflammatory peptides, polyketides, and phenol-derivatives [1]. A summary of the different strains with anti-inflammatory activities is provided in Table 2.

According to the bibliography, most of the marine species with anti-inflammatory activity belong to the *Aspergillus* (41.4%) and *Penicillium* (27.1%) genus. These marine genera produce anti-inflammatory agents of various structure types—alkaloids, terpenoids, polyketides, peptides, etc. [136]. Preussins from *Aspergillus flocculosus* 16D-1, isolated from a marine sponge, displayed even stronger inhibition of IL-6 expression than those of the positive control [115]. *A. terreus* was reported to inhibit NO due to the content of alkaloids, terpenoids, peptides, and polyketides. Among them, a rare natural compound with an unusual structure ((*E*)-oxime group) was isolated. The substances with anti-inflammatory activities against NO production from *A. terreus* demonstrated serious inhibitory potential with IC_50_ values in the range from 5.48 to 29.34 μM [116]. *A. versicolor* was shown to possess an alkaloid (asperversiamide G) which acts against iNOs with an IC_50_ value of 5.39 μM [118]. *A. terreus* CFCC 81836 and *Aspergillus* sp. SCSIOW2 exerted moderate anti-inflammatory activity through NO inhibition thanks to the terpenoid content. *A. terreus* CFCC 81836 was a source of brasilanones A and E that manifested NO inhibition rates of 47.7% and 37.3%, respectively, at the concentration of 40 μM [117]. Dihydrobipolaroxins from *Aspergillus* sp. SCSIOW2 exerted inhibitory effects on NO production induced by LPS/INF-γ in a dose-dependent manner [119]. Polyketides from *A. niger* SCSIO Jcsw6F30 and *Aspergillus* sp. SCSIO Ind09F01 acted against COX-2. Aurasperones F, C, and A from *A. niger* SCSIO Jcsw6F30 inhibited COX-2 with IC_50_ values of 11.1, 4.2, and 6.4 μM, respectively, while compounds 7, 8, and 11 from *Aspergillus* sp. SCSIO Ind09F01 inhibited the enzyme with IC_50_ values of 2.4, 7.1, and 10.6 μM, respectively [120,121]. The peptides violaceotide A and diketopiperazine dimer from *A. violaceofuscus* were found to act against the IL-10 expression with the rate of inhibition 84.3% and 78.1% at 10 µM in LPS-activated THP-1 cells [122].

The alkaloids viridicatol, brevicompanines E and H, and methylpenicinoline from several *Penicillium* species were the reasons for the NO inhibition [137,138,139]. Viridicatol, which is a quinolone alkaloid, suppressed the iNOS and COX-2 expression and further inhibited iNOS-derived NO and COX-2-derived prostaglandin E2 (PGE_2_) in LPS-stimulated RAW264.7 and BV2 cells. In addition, viridicatol inhibited the expression of the pro-inflammatory cytokines IL-1β, IL-6, and TNF-α. In subsequent analysis, it was revealed that this alkaloid inhibited the nuclear factor-kappa B pathway in LPS-stimulated RAW264.7 and BV2 cells. The phosphorylation and degradation of inhibitor kappa B-α in the cytoplasm was blocked, and the translocation of NF-κ B p65 and p50 heterodimer in the nucleus was suppressed [137]. The brevicompanines E and H inhibited the LPS-induced NO production in BV2 microglial cells [138]. Methylpenicinoline was found to suppress the iNOS expression in RAW264.7 macrophages and BV2 microglia, thus inhibiting NO production. It also minimized the production of prostaglandin E_2_ through the suppression of COX-2 expression in a concentration-dependent manner (10 μM—80 μM) [139]. Thomimarine E from *P. thomii* showed activity against NO with a 22.5% inhibition rate at 10.0 µM in LPS-activated RAW264.7 cells [123].

Several compounds with anti-inflammatory activity were illuminated in the marine-derived fungi *Penicillium glabrum* (SF-7123), namely neuchromenin, myxotrichin C, and deoxyfunicone. These metabolites demonstrated inhibitory activity against the overproduction of NO in LPS-stimulated BV2 microglial cells with IC_50_ values of 2.7 µM, 28.1 µM, and 10.6 µM, respectively. The excessive production of NO in LPS-stimulated RAW 264.7 macrophage cells with IC_50_ values of 4.7 µM, 41.5 µM, and 40.1 µM, respectively, was also inhibited. Moreover, these compounds inhibited LPS-induced overproduction of prostaglandin E_2_. The most active metabolite (neuchromenin) passed through a further investigation which revealed that the anti-inflammatory activity was related to the suppressive effect on the overproduction of inducible NO synthase and COX-2 enzymes. It was also established that these effects were mediated through the downregulation of inflammation-associated pathways such as those dependent on NF-kB and p38 mitogen-activated protein kinase in LPS-stimulated BV2 and RAW 264.7 cells [125].

*Bacillus liquefaciens* M116 was recently isolated from sediments of the south coasts of the Cuban platform. The extract obtained from its fermented broth proved to contain anti-inflammatory compounds. It inhibited acute inflammation in Croton oil-induced atrial edema in mice. Croton oil induces the typical phases of acute inflammation. The single oral dose of M116 extract for acute inflammation reduction was 50 ÷ 200 mg/kg. Inhibition of chronic inflammation in cotton pellet-induced granuloma in Balb/c mice was also achieved [126]. In a study by Abdel-Wahab et al., it was suggested that the anti-inflammatory properties of other *Bacillus* strains are owing to the ability to inhibit the activities of the enzymes LOX and COX [127].

Melanin from marine *Bacillus* spp. BTCZ31 was reported to exert anti-inflammatory properties [128]. Melanins are brown complex pigments that are produced via the amino acid tyrosine and are responsible for various biological functions, such as thermoregulation, cation chelators, photoprotection, free radical sinks, and antibiotics. In humans, besides the determination of skin color, melanin protects against UV radiation. In microorganisms, this pigment prevents environmental stresses. Melanins are known to possess antioxidant properties as they reduce ROS generation [140]. The reduction in ROS minimizes inflammation, as well. Kurian and co-authors established that melanin from *Bacillus* spp. BTCZ31 inhibited COX and LOX enzymes effectively at increasing concentrations. The COX enzyme was inhibited with an IC_50_ value of 104.34 µg/mL, while in contrast, the LOX enzyme was inhibited with an IC_50_ value of 10.5 µg/mL. It was calculated that 100 µg/mL of BTCZ31 melanin inhibited COX and LOX enzymes at 47.92 and 69.48%, respectively. Moreover, cellular nitrite levels, an NO indicator produced during inflammation, reduced with the increase in melanin concentration. These results altogether prove the potential of melanin as an anti-inflammatory agent [128].

Yellow-pigmented *Micrococcus* sp. was demonstrated to exhibit anti-inflammatory activity. The pigment was extracted with methanol from the bacterial pellet. The conducted in vivo assays with rats resulted in higher collagen content, granulation tissue formation, and increased migration of macrophages and fibroblasts cells at the site of the wound. The anti-inflammatory properties of the extracted pigment were developed using the carrageenan-induced rat paw edema. Carrageenan-induced edema is biphasic: in the first phase, serotonin and histamine are released, and in the second phase, they are mediated by prostaglandins and cyclooxygenase products. The application of 10% pigment resulted in reduced wound closure time. The results of this study show that the yellow pigment from *Micrococcus* is a promising option for wound healing treatment [129]. The team of Srilekha tested another marine bright yellow-pigmented bacteria identified as *Brevibacterium* sp. [130]. They analyzed the pigment extract in vivo and it was revealed that it possessed effective anti-inflammatory properties. Microbial pigments are a scientific target as they are natural and safe, they are characterized by medicinal properties, vitamin composition, production independent of the season and geographical conditions, and a controllable and predictable yield. Furthermore, natural colorants are believed to be non-toxic, non-carcinogenic, and biodegradable [141].

*Eurotium amstelodami* was isolated from marine materials. The anti-inflammatory assay with its broth and its mycelium extracts resulted in the inhibition of NO production in LPS-stimulated RAW 264.7 cells without cytotoxicity. Further, several components were extracted including asperflavin, neoechinulin A, and pre-echinulin. Asperflavin markedly inhibited LPS-induced NO and PGE2 production in a dose-dependent manner. Asperflavin inhibited the production of TNF-α, IL-1β, and IL-6 as well [131].

Various marine microorganisms synthesize fatty acids. Fatty acids can be separated into two major groups—saturated and unsaturated. Unsaturated fatty acids can be mono-, di-, and polyunsaturated fatty acids (PUFAs). Omega 3 (n-3) and omega 6 (n-6) are examples of PUFAs with pronounced anti-inflammatory activity [142]. However, the human organism is incapable of synthesizing them. Their function in regulating inflammation is very important. The polyunsaturated fatty acids (PUFAs) omega 6, like arachidonic acid (ARA), promote inflammation while omega 3 fatty acids like eicosapentaenoic (EPA) and docosahexaenoic (DHA) acid exhibit anti-inflammatory properties. Omega 3 fatty acids suppress inflammation through different pathways. On one side, they inhibit omega 6 fatty acid-derived pro-inflammatory eicosanoids (prostaglandin E, leukotriene B4) formation. On the other side, omega 3 can create diverse potent anti-inflammatory lipid mediators (such as resolvins and protectins) [142]. EPA is associated with important physiological functions including the cardiovascular system and blood pressure, it cleanses the arteries, and diabetes, treats atherosclerosis, and suppresses the inflammatory systems. ARA is the most important PUFA for normal brain function. Both EPA and ARA were reported to be produced by the red marine microalgae *Porphyridium* sp. *Porphyridium* possesses GRAS status [134]. In a recent paper, high amounts of ARA from *Porphyridium cruentum* were reported [143]. Microalgae are an important constituent of the human diet for their nutritional value and the rich protein content, which is higher than the protein content of some vegetables [144]. EPA was shown to be produced by the marine bacteria *Vibrio cyclitrophicus* [132]. Producers of EPA and DHA are reported among the strains of *Cellulophaga* and *Pibocella* [133].

The marine actinomycete *Streptomyces* are known to produce a wide range of secondary metabolites with potent biological activities [135]. The team of Shin isolated four new streptoglycerides E-H (1–4) from *Streptomyces specialis* with a rare 6/5/5/-membered ring system. All of the compounds displayed moderate anti-inflammatory effects with IC_50_ values in the range of 3.5–10.9 µM. The analysis showed that compound 2 inhibited the mRNA expression of iNOs and IL-6 in RAW 264.7 cells without cytotoxicity observed. NO_2_^−^ accumulation was used as a criterion for NO production in media [145]. These streptoglycerides are promising bioactives with good anti-inflammatory activity with potential for further studies for their therapeutic use.

## 6. Conclusions

Inflammation is responsible for a broad spectrum of pathophysiological dispositions ranging from acute and chronic infections and cancer to autoimmune-based conditions such as those in the gastrointestinal tract. Targeting reductions in chronic inflammation to normal levels is key to striving with associated diseases. Although the medicines that are applied in these cases (steroidal and non-steroidal drugs) suppress inflammation, they are not a good alternative in the long term due to the side effects associated with their use (liver and kidney dysfunction, intestinal problems, disorders in the cardiovascular and endocrine systems, etc.).

Nature provides unlimited sources of pharmaceutical agents. The employment of beneficial microorganisms has repeatedly proven its merits in various socially significant diseases. A plethora of microbes with anti-inflammatory effects between bacteria, yeast, and fungi living in different habitats have been reported in recent years. These microorganisms produce specific metabolites which suppress the over-production of cytokines and/or the continuous secretion of pro-inflammatory cytokines, thus providing an anti-inflammatory effect. Most of them are found in the lactobacilli family or consortia with them. As a form of adjuvant therapy, probiotic microorganisms are used to alleviate patients’ symptoms, and promising effects in the case of inflammatory diseases have been shown. The probiotics are characterized by a limited number of side effects which are contributed by their pleiotropic immune modulatory behavior. They are commonly included in functional foods due to their GRAS status and diverse benefits for human health. On the other hand, marine microorganisms are one more valuable renewable resource with proven bioactive characteristics including anti-inflammatory properties. Given their ability to survive under extreme conditions, they are expected to synthesize a wide variety of substances with incomparable chemical diversity and drug-like effects.

Counteracting chronic inflammation through natural drug sources is characterized by multiple advantages, including their renewable and inexhaustible nature, the fact that they are non- or low-toxic, and the lack of side effects. The production of metabolites with anti-inflammatory properties could be enhanced by varying the culture conditions.

Although a significant body of knowledge has been accumulated, regarding the limitless variety of microorganisms in nature, still a large number of representatives with anti-inflammatory properties have not been fully recognized and appreciated. This fact opens an avenue for the future exploration of newly isolated strains as well as optimizing their growth parameters for maximized anti-inflammatory substance production. The opportunity to discover and utilize new drug sources and to optimize their isolation and purification still lies ahead of researchers.

## Figures and Tables

**Table 1:** LAB with anti-inflammatory activity.

Strain	Activity	Reference
*L. reuteri* DSM 17938 (lysate)	Decreased levels of IL-6 and IL-8	[19]
*L. reuteri* ATCC PTA6475	Synthesizes folate; suppression of TNF-α production in human monocytes	[53]
*L. reuteri* MG9012	Reduced NO production	[54]
*L. paracasei* CBA L74		
*L. brevis*	GABA production; inhibition of NO and iNOs production, and NF-kB activity	[55]
*L. fermentum* MG9014	Reduced NO production	[54]
*L. plantarum* CRL2130	Produced riboflavin; intestinal inflammation reduction via pro-inflammatory cytokines control	[56]
*L. plantarum* OLL 2712	Induced activity of IL-10	[57]
*L. plantarum* M2, *L. plantarum* K09	*TNF-α suppression*	[58]
*L. paraplantarum* BGCG11	EPS production; decreased levels IL-1β, TNF-α, and iNOS, and enhanced levels IL-10 and IL-6	[59]
*L. rhamnosus* RW-9595M	EPS production; IL-10 production inhibition	[60]
*Lactobacillus rhamnosus*	Reduced levels of IL-6 and C Reactive Protein	[61]
*L. intestinalis* LE1 and *L. johnsonii* LE2	Reduced in vitro mercury toxicity on the intestinal mucosa	[62]
*L. plantarum* SGL 07, *L. salivarius* SGL 19 (lysates)	Stimulation of keratinocytes proliferation	[20]
*L. plantarum* CRL2130, *Streptococcus thermophilus* CRL807, and *Streptococcus thermophilus* CRL808 (blend)	Riboflavin, folate production, immune-modulatory properties; decreased levels of IL-6, increase in TNF-α	[63]
*L. casei*, *L. plantarum*, *L. acidophilus*, *L. delbrueckii* subsp. *bulgaricus*, *Bifidobacterium (B.) longum*, *B. breve*, *B. infantis*, and *S. salivarius* (blend)	Decreased levels of TNF-α and IL-6 in colon tissue	[64]
*Lactobacillus*, *Lactococcus*, *Leuconostoc*, *Streptococcus*, *Acetobacter*, *Kluyveromyces*, *Torula*, *Candida*, *Saccharomyces* (kefir consortium)	Kefiran production; induced CD^4+^ and CD^8+^ T-lymphocytes populations	[65]
	Kefiran production; normalized levels of IL4 and IL5 levels	[66]
*L. casei*, *L. acidophilus*, *Lactococcus lactis*, *Leuconostoc citrovorum*, *L. mesenteroides*, *Acetobacter aceti*, *A. rasens*, *Streptococcus thermophilus*, *S. lactis*, *Kluyveromuces* sp., *Sacharomyces* sp. (Tibetan mushroom consortia)	Granuloma formation inhibition	[67]
*Lactobacillus* sp., *Acetobacter xylinoides*, *Gluconobacter oxydans*, *Komagataeibacter xylinum*, *Gluconacetobacter hansenii*, *Oenococcus oeni*, *Komagataeibacter europaeus*, *Schizosaccharomyces pombe*, *Zygosaccharomyces kombuchaensis*, *Torulaspora delbrueckii*, *Saccharomyces* sp., *Brettanomyces* sp. (kombucha consortia)	Riboflavin production; 87–91% improved anti-inflammatory activity; IC_50_ value close to the maximal inhibitory concentration of nordihydroguaiaretic acid	[68]
*Leuconostoc mesenteroides* BioE-LMD, *Bacillus licheniformis* BioE-BL11 (isolated from Korean kimchi)	EPS production; inhibited secretion of IL-6; increased secretion of IL-10	[69]
*L. plantarum* LM17 and LM19, *L. rhamnosus* LM07 (agave fermentation stage)	Decreased intestinal permeability	[70]
*L. casei* EMRO 002, *L. casei* EMRO 213, *L. plantarum* EMRO 009, *L. fermentum* EMRO 211, *L. rhamnosus* EMRO 014, *L. bulgaricus* EMRO 212, *Rhodopseudomonas palustris* EMRO 201 (multi-strain extract)	Inhibition of migration inhibitory factor tautomerase activity	[71]
*L. mucosae* AN1, *L. fermentum* SNR1 (encapsulated)	Anti-inflammatory cytokines upregulation and pro-inflammatory cytokines downregulation	[49]

**Table 2:** Marine microbes with anti-inflammatory properties.

Strain	Activity	Reference
*A. flocculosus* 16D-1	Inhibition of IL-6 expression	[115]
*A. terreus*	NO inhibition	[116]
*A. terreus* CFCC 81836	NO inhibition	[117]
*A. versicolor*	Possession of alkaloids that act against iNOs	[118]
*Aspergillus* sp. SCSIOW2	NO inhibition	[119]
*A. niger* SCSIO Jcsw6F30	Act against COX-2	[120]
*Aspergillus* sp. SCSIO Ind09F01	Act against COX-2	[121]
*A. violaceofuscus*	Act against IL-10 expression	[122]
*P. thomii*	NO inhibition	[123]
*Penicillium atrovenetum*	Possession of anti-neuroinflammatory meroterpenoid citreohybridonol	[124]
*Penicillium glabrum* (SF-7123)	NO inhibition	[125]
*Bacillus liquefaciens* M116	Granuloma reduction	[126]
*Bacillus* sp.	Inhibition of the activities of LOX and COX enzymes	[127]
*Bacillus* spp. BTCZ31	Melanin production;COX and LOX inhibition	[128]
*Micrococcus* sp.	Produces yellow pigment reducing the wound closure period	[129]
*Brevibacterium* sp.	Anti-inflammatory activity comparable to diclofenac	[130]
*Eurotium amstelodami*	Produces asperflavin; inhibition of LPS-induced NO, PGE2, TNF-α, IL-1β, and IL-6 production;	[131]
*Vibrio cyclitrophicus*	EPA production	[132]
*Cellulophaga*	EPA and DHA production	[133]
*Pibocella*	EPA and DHA production	[133]
*Porphyridium* sp.	ARA production	[134]
*Streptomyces specialis*	Inhibition of mRNA expression of iNOs and IL-6	[135]
